# Perceptions and Attitudes of Health Professionals in Kenya on National Health Care Resource Allocation Mechanisms: A Structural Equation Modeling

**DOI:** 10.1371/journal.pone.0127160

**Published:** 2015-06-03

**Authors:** Patrick Opiyo Owili, Yi-Hsin Elsa Hsu, Jin-Yuan Chern, Chiung-Hsuan Megan Chiu, Bill Wang, Kuo-Cherh Huang, Miriam Adoyo Muga

**Affiliations:** 1 School of Health Care Administration, Taipei Medical University, Taipei, Taiwan; 2 International Health Program, Institute of Public Health, National Yang Ming University, Taipei, Taiwan; 3 Department of Health Care Administration, Chang Jung Christian University, Tainan, Taiwan; 4 Department of Health Care Administration, Asia University, Taichung, Taiwan; 5 Institute of Community Health and Development, Great Lakes University of Kisumu, Kisumu, Kenya; Örebro University, SWEDEN

## Abstract

**Background:**

Health care resource allocation is key towards attaining equity in the health system. However, health professionals’ perceived impact and attitude towards health care resource allocation in Sub-Saharan Africa is unknown; furthermore, they occupy a position which makes them notice the impact of different policies in their health system. This study explored perceptions and attitudes of health professionals in Kenya on health care resource allocation mechanism.

**Method:**

We conducted a survey of a representative sample of 341 health professionals in Moi Teaching and Referral Hospital from February to April 2012, consisting of over 3000 employees. We assessed health professionals’ perceived impact and attitudes on health care resource allocation mechanism in Kenya. We used structural equation modeling and applied a Confirmatory Factor Analysis using Robust Maximum Likelihood estimation procedure to test the hypothesized model.

**Results:**

We found that the allocation mechanism was negatively associated with their perceived positive impact (-1.04, *p* < .001), health professionals’ satisfaction (-0.24, *p* < .01), and professionals’ attitudes (-1.55, *p* < .001) while it was positively associated with perceived negative impact (1.14, *p* < .001). Perceived positive impact of the allocation mechanism was negatively associated with their overall satisfaction (-0.08) and attitude (-0.98) at *p* < .001, respectively. Furthermore, overall satisfaction was negatively associated with attitude (-1.10, *p* <.001). On the other hand, perceived negative impact of the allocation was positively associated with overall satisfaction (0.29, *p* <.001) but was not associated with attitude.

**Conclusion:**

The result suggests that health care resource allocation mechanism has a negative effect towards perceptions, attitudes and overall satisfaction of health professionals who are at the frontline in health care. These findings can serve as a crucial reference for policymakers as the Kenyan health system move towards devolving the system of governance.

## Introduction

Health professionals in Sub-Saharan Africa are always facing challenges of scarce health care resources which is leading to rationalization health care standards [1]. Today, health care resource allocation and equity is very important towards attaining efficiency in the health system. However, health professionals have reported the existence of different levels of resource availability, perceived health care equity, and discrimination which leads to preferences in the allocation and distribution of health care resources [2, 3].

Kenya is not an exception when it comes to allocation of health care resources. It is among the poorest countries in the world and was ranked 154 out of 177 countries on the United Nations Development Programme (UNDP) Human Development Index for 2005 and about 22.8% survive on a dollar or less per day while 58.3% survive on less than two dollars per day [4]. Nearly 40% of its estimated 40 million population are unemployed [5]. Politically, it has been a relatively stable country in the region of East Africa. It has a functioning multi-party democracy and has manifested economic growth in recent years, to be ranked 128 out of 177 countries by UNDP in 2011 [6]. Like many other countries in sub-Saharan Africa, Kenya faces serious health challenges. Non-communicable diseases such as diabetes disease are on the rise, but the leading causes of death still remain poverty related diseases, notably malaria [7].

Currently, Kenya’s health system focuses on coordination, overall guidance, strategic planning, and policy formulation of the sector [8]. The country’s 6-tier health system—which had level 1 (The community units), level 2 (The dispensaries or basic health facilities), level 3 (The health centers), level 4 (The district level), level 5 (Provincial) and level 6 (National)—is currently being reorganized towards a devolved system of governance. In 1989, user fees or ‘cost-sharing’ was introduced and later abolished for outpatient care in 1990 after inspiration by concerns of social justice but was re-introduced in 1992 because of budgetary constraints. To date, these fees have remained and have impacted access to health care negatively [9, 10].

Enhancing equitable access to quality health care still remains the major objective of health sector reform as outlined in Kenya’s Vision 2030 and the Medium Term Plan 2008–2012 [11, 12]. Unfortunately, it remains elusive, primarily as a result of the health care financing system, high level of poverty and lack of financial access. About 40% of the sick people who did not seek health care services cited inability to pay as the main reason, and hence, lack of finance become a major obstacle to improving the populace health. Though, several attempts have been made to review health care financing system and to abolish fees in any form within the public sector, it has met resistance from various groups [13]. However, the government has continued to search for sustainable solutions to health care financing in the country. This kind of scenario makes access to health care a big problem for the majority of people living below the national poverty line (less than $1 a day), that constitute about 45.9% of the population in Kenya as per 2005/2006 statistics [7].

### Health care resource allocation

At the very initial stages national resource allocation in the public sector was on an incremental basis where government departments would get a fixed raise each year proportional to the Treasury’s anticipated volume of resources. Moreover, this model of budgeting neglected the three stages of budgeting and resource allocation (analyzing, fitting, and implementing) where the fitting stage (reducing budgets to ceilings) was overemphasized and then analyzing and the implementing stages were neglected and thus, it lacked mechanisms for promoting analysis [14]. Resource allocation was then transformed to consider disparities in regions and sectors, by introducing a three-year rolling and forward budget framework which still remained largely devoid of needs-based criteria. At the end of the 1990s, Medium-Term Expenditure Framework (MTEF) approach to budgeting at national level along and poverty reduction strategic planning was linked to proposed outputs involving three-year spending cycles in which the last two years included activities designed to support and bolster the activities of the first budget year [15]. Treasury department would then set expenditure ceilings for Sector Working Groups (SWGs) of stakeholders who would agree on budget allocations within the group keeping in mind sector’s priorities from the action plans and the national development plan. The presumption was that membership in these SWGs should be broad enough to give a voice to national and sub-national priorities. In reality, however, information from the sub-sectors and the sub-national level did not flow well to the SWGs, and the sub-national agendas often were ignored. Consequently, sub-sectors groups which did not have strong political lobbies were unlikely to influence SWG allocation decisions and hence ministries invariably failed to obtain requested resources which undermined the implementation of their planned activities [16].

In 2008, Ministry of Health (MOH) in Kenya was split into two separate ministries with one ministry focusing on curative services and the other focusing on preventive and promotive health care, which made tracking of health expenditure somewhat complicated. The two ministries were depending on the Ministerial Budgeting Committee (MBC) which was attended by all department heads who in turn would bring budgetary proposals, and then would rely on persuasion, political influence and goodwill of other departments to allocate MOH’s limited resources. Only a small portion of resources would then be allocated on a more objective and measurable basis through a Resource Allocation Criteria (RAC) formula which was still being implemented at the district level and rural health facilities by use of a weighted average that considers poverty rate, bed use, outpatient case load among others as presented in S1 Table [16]. Moreover, the health budget allocation has continued to be skewed in favor of tertiary and secondary care facilities, which absorb 70% of health expenditures [17]. Yet primary care units, the first line of contact with the population, provide the bulk of health services and are cost effective in dealing with the disease conditions prevalent in communities. Today, it is not known what will define health care system with a devolved system of governance in place in Kenya.

El-Ashry and Gibbons [18] identified seven important characteristics which are found, to a large degree, in market processes for the allocation of scarce resources which includes: (1) Flexibility in the allocation, (2) security of tenure for established users, (3) voluntary response by user to incentives for allocating resources, (4) allocation mechanisms should confront the user with real opportunity cost of the resources being used and with that, only economically efficient mechanism will be chosen, (5) allocation process should be perceived by the public as equitable and fair, (6) socially responsible allocation process must reflect public values that may not be adequately considered by individual users and (7) it is desirable that the outcome of the allocation process be predictable in particular applications, and that the allocation mechanisms should incorporate some degree of uncertainty. It is, however, not known if the Kenyan health allocation criteria possess these characteristics. Guindo, Wagner [19] also identified many factors, from health benefits to the overall context of health system, which require careful consideration by health policy decision makers when allocating health care resources.

Equity in health is one of the central reasons for allocating health care resources. The poor people continue to bear the greatest burden of disease, but receive a smaller portion of health care resources than do the healthy and better-off. This phenomenon known as “the inverse care law” in which health care resources are distributed inversely in relation to need [20]. Inequalities in health are preventable to the extent that they stem from discernable policy decisions exercised by governments, such as tax policy, health care funding and priority setting in the distribution of health care resources [21]. Other concerns for allocating health care resources includes infrastructure development [22], cost and clinical effectiveness [23], and health benefits as well other non-health benefits such as economic benefits among others [19, 24]. This study, however, considered four aspects which are among the concerns of resource allocation, and this includes equity as a result of fair distribution of resources, clinical effectiveness, resulting from the human resource availability, economic impact owing to improved income and standards of living as well as risk pooling, and infrastructure development as a result acquisition of equipment and availability of facilities.

Inequality in health care resource allocation is still an issue of concern to health professionals in Kenya even though its limited resources are being distributed using MTEF and RAC approaches. These professionals occupy a very important position which makes them notice the impact of the changes set by their health care system, in addition to confrontation with the effects of health care resource allocation mechanism on their clinical practice, forcing them to focus more on health care rationing. In recent years, the health sector in Kenya was marred with a lot of strikes from health professionals. Moi Teaching and Referral Hospital (MTRH) was faced with financial challenges that saw at least 130 doctors and nurses resign in the months of July to September, 2011 citing low morale and lack of resources as the main reason they are seeking alternative employment [25]. This was coupled with a nationwide strike of all health professionals that crippled services in public hospitals [26, 27]. National health care resource allocation mechanism, though appropriate, has some policies that may limit and restrict health professionals in the performance of their duties, especially on exercising their commitment to offering the best possible treatment to their patients within a resource constrained setting.

Even though low-income countries are still faced with challenges of inequalities in access to health care, poor data collection and availability, as well as severe budgetary constraints, health professionals still have an ethical responsibility to offer the best available medical care to their patients even if this responsibility conflicts with their role as gatekeepers of the limited health care resources which are available collectively for all patients. Therefore, by exploring the perceptions and attitudes of health professionals, who witness the effects of different health care resource allocation mechanisms in Kenya at the frontline, it provides a useful insight regarding priority setting on allocation of health care resource in low-income countries which are concerned with improving the health allocation mechanism to enable health professionals to perform their duties without restrictions or rationing.

However, health professionals, who are still at the frontline of the health care system, occupy a position which makes them notice the impacts of policies set by their health care system, and are quite often confronted with the effects of national health care resource allocation mechanism on their clinical practice. Studies have been conducted in Kenya on perceptions and attitudes of health professionals on research location impact, mental health policy, emergency contraception, and the resident’s experience of the medical profession [28–31]. But, there remain no documented studies in Kenya on perceptions and attitudes of health professionals regarding health care resource allocation mechanism. This study, therefore, seeks to determine perceptions and attitudes of health professionals in Kenya regarding national health care resource allocation mechanism in Kenya, using Structural Equation Modeling (SEM).

## Methods

### Conceptual model

**Fig 1:**
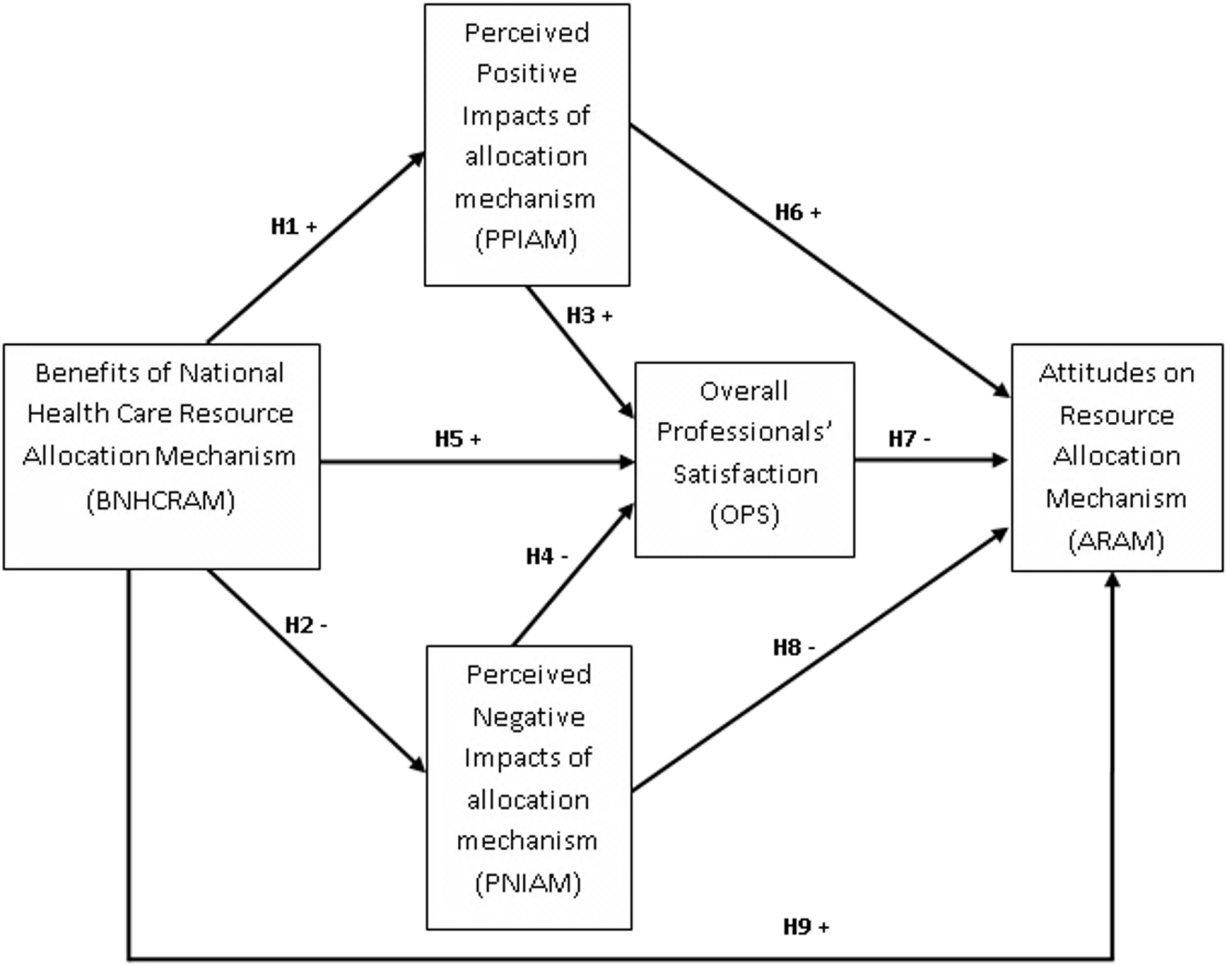
Hypothesized structural relationships of perceived impacts and attitudes towards allocation mechanism.

Since researchers are encouraged to identify both the competing theoretical models against which the fit of the model of interest can be compared and the equivalent models, this study adapted a hypothetical model (Fig 1) that studied perceptions and attitudes in Tourism [32–35]. The variables were adjusted to reflect the important aspects of national health care resource allocation mechanism and health professionals. The hypothetical model has nine path hypotheses, which are the relationships among five latent constructs: benefits of national health care resource allocation mechanism, perceived positive impacts of allocation mechanism, perceived negative impacts of allocation mechanism, overall health professionals’ satisfaction, and attitudes on resource allocation mechanism. Each path represents a hypothesized relationship with the direction of effect identified as either positive (+) or negative (–). This research tested the goodness-of-fit of the model and hypotheses with SEM.

### Setting and ethical statement

The study was undertaken in Moi Teaching and Referral Hospital (MTRH) with 3,066 employees of varied specialties and with vast experience in both medical practice and teaching in Kenya, it is located in the town of Eldoret and serves 40% of the Kenyan population with its 560 bed capacity [36]. Kenya has only two national hospitals of which MTRH is one of the two. Approval to conduct the study was sought from Institutional Research and Ethics Committee (IREC) of MTRH (IREC/2011/187) before data collection, and this enhanced mobilization of the targeted population. Informed consent was obtained from the participants before self-administration of the questionnaire.

### Instrument, sampling procedure and data collection

A 5-point Likert type scale (5 = strongly agree; 1 = strongly disagree) was developed, before an Instrument-Content Validity Index (I-CVI) was performed to determine the importance, appropriateness and clarity of each questionnaire item. There is no firm guideline as to the relative benefit of one type of scale over the other and therefore, the selection of a 5-point scale instead of 7-point or 10-point scales is based largely on the degree maturity, as the 10-point scale permits further granularity to be extracted from the data. Therefore, the 5-point scale was adopted for this study due to its simplicity for the respondent and the ease of use.

Since a new scale was developed, it was important that the scale and the items on it to be evaluated if the content are valid. I-CVI was then performed on the questionnaire items by three experts who rated each questionnaire item by considering three things—importance, appropriateness and clarity of each question. Content validity concerns the degree to which a scale has an appropriate sample of items to represent the construct of interest—that is, whether the domain of content for the construct is adequately represented by the items [37]. A Content Validity Index (CVI) value is computed for each item on a scale as well as for the overall scale. To calculate an item-level CVI, the three experts were asked to rate the relevance of each item on a modified ordinal 5-point scale of 1 = not relevant, 2 = a little relevant, 3 = relevant, 4 = more relevant, 5 = highly relevant. The score for each item was then summed up and averaged out based on the total expected score (15) to get the index, and finally obtaining the overall I-CVI by summing up all the scores of all questionnaire items and getting the overall average. I-CVI of 0.78 or higher for three or more experts is considered evidence of good content validity [38]. The overall I-CVI score of our instrument was 0.92 after the deletion of items below a score of 0.78. The final survey questionnaire which had four sections was used to assess perceived impact of resource allocation mechanism and attitudes of health professionals (See S1 Appendix for the final questionnaire).

The first section of the questionnaire included some questions on socio-demographic characteristics of the participant, consisting of years of experience and specialty. The second section consisted of questions regarding the perceived impact of the health care resource allocation. The third section had questions about overall satisfaction of health professionals while the final section had questions asking opinion on health care resource allocation mechanism and their attitude.

In this study, Perceived positive and negative impact of the allocation mechanism had items which measured four indicators (Equity, development, efficiency and economic impact) while overall health professionals’ satisfaction had items measuring satisfaction with health care provided to the population, their job, as well as questions on equity, efficiency and economic impact. Health professional attitude towards health care, development, equity, and allocation mechanism. Questions regarding health care resource allocation mechanism considered eight characteristics: complexity, meeting health care needs, encouraging efficiency, improving equity in health care, catering for the unmet needs, and encouraging development and hospital performance.

Sample size calculation software from Demetra’s website was used to determine the minimum required sample size [39]. A 5% margin of error and the 95% confidence level was adopted to obtain the required study sample from a total population of 3,066 employees at MTRH, and a sample of 341 was determined before a cross-sectional administration of the questionnaire in each department. Enumerators were selected among medical students to participate in the data collection process. They were then trained on data collection procedure using the questionnaire. Data collection was supervised closely and verified if respondents answered all the questionnaire items before data entry procedures were followed. All the data used in this study were anonymized. The targeted respondents were asked if they were willing to participate, and if they agreed, they would then be given the questionnaire to answer the questions. Enumerators would monitor and help in the clarification of any part of the questionnaire if they did not understand. But, if someone was not willing to participate, the enumerators would apologize and leave. The targeted respondents were clearly informed of the voluntary nature of their participation and could decline their consent at any time. A total of 325 self-administered questionnaires were returned but due to missing data in 25 cases, 300 cases were used in the final analysis and hence 87.9% response rate was attained. Moreover, in examination of published SEM research, Schumacker and Lomax [40] found that many articles used from 250 to 500 subjects, although the greater the sample size, the more likely that one can validate the model using cross-validation, they also found that others were in agreement that 100 to 150 subjects is the minimum satisfactory sample size when conducting SEM. Having collected enough data that was analyzable using SEM method, data were then entered and verified in MS Excel before performing two stages of the analysis.

### Reliability analysis and measurement variables

The measurement items were assessed for a convergent validity by ensuring all items load high on one factor. This reliability analysis was used to evaluate stability and consistency of the measurement items for each latent construct. The criteria used in deciding whether to delete an item was its corrected item-to-total correlation and alpha if item deleted. In general, items with corrected item-to-total correlations below 0.30 should be eliminated [41, 42].

Cronbach’s alpha reliability coefficient usually ranges between 0 and 1. Nevertheless, there is really no lower limit to the coefficient. The nearer Cronbach’s alpha coefficient is to 1.0 the greater the internal consistency of the items in the scale. George and Mallery [43] provided the following easy rule of thumb to interpreting Cronbach alpha: “_ > 0.9—Excellent, _ > 0.8—Good, _ > 0.7—Acceptable, _ > 0.6—Questionable, _ > 0.5—Poor, and _ < 0.5—Unacceptable”. It should be noted that an alpha of 0.7 and above is doubtlessly a reasonable goal [41]. While a "high" value for Cronbach alpha indicates good internal consistency of the items in the scale, it does not infer that the measure is unidimensional. Factor analysis is one method of checking dimensionality of a scale. In other words, Cronbach's alpha is not a statistical test—It is a coefficient of internal consistency (or reliability). After a reliability analysis, the measurement items were revised down as indicated in S2 Table.

‘Perceived positive impacts of the allocation mechanism’ had seven items with a Cronbach-α Coefficient of 0.84. However, since four indicators were measured, the indicators with the highest corrected item-total correlation in each category were used in the final analysis. After deletion of the other items, the Cronbach-α declined to 0.80 but exceeded the recommended level of 0.70 [41]. ‘Perceived negative impacts of the allocation mechanism’ had seventeen items with a Cronbach-α Coefficient of 0.62. After deletion of the other items that did not meet the threshold, the Cronbach-α of the four measured items rose to 0.82 which was also above the recommended level.

‘Overall health professionals’ satisfaction’ had eleven items with a Cronbach-α Coefficient of 0.85. But, after deletion of the other items that were below the required level, the Cronbach-α of the six items remained constant at 0.85 which was above the recommended level of 0.70. ‘Attitude on resource allocation mechanism’ had 15 items with a Cronbach-α Coefficient of 0.81 in this construct. However, deletion of the items below the required level made the Cronbach-α of the five items to rise above the recommended level at 0.86.

‘Benefits of national health care resource allocation mechanism’ had eight items with a Cronbach-α Coefficient of 0.77. And by the deletion of one item below the minimum level, the Cronbach-α of the remaining seven items rose to 0.82 which exceeding the recommended level of 0.70.

The final measures for perceived positive and negative impacts included four measurement variables each, which addressed four aspects: equity, development, efficiency, economic growth concerns. A code (in parenthesis) was assigned to each item and was used for easy generation of the path diagram. The latent construct, *Perceived positive impacts of the allocation* (PPIAM) included fair distribution of resources (Peq2_13b), acquisition of modern equipment (Pde2_3), increase in human resource (Pef2_15), and improved income and standards of living (Pec2_16). On the other hand, *Perceived negative impacts of the allocation* (PNIAM) construct included increased cost of care (Neq2_12e), inadequate facilities (Nde2_7), increased hospital length-of-stay (Nef2_6), and inability to attain universal coverage (Nec2_12d).

*Overall health professionals’ satisfaction* (OPS) included six variables which measured satisfaction with patients’ services offered (Shc3_1b), resources available (Sjo3_2a), the information available (Sjo3_2c), cost of services (Seq3_3a), hospital performance (Sef3_3c), and income and standards of living (Sec3_3d). *Attitude on allocation of health care resources* (ARAM) included whether the allocation criteria should consider patient volume (Ah4_9b), improvement in facilities and equipment (Ade4_11b), increase in human resource (Aec4_11a), patients’ service restrictions (Aeq4_9e), and hospital size (Aal4_10d).

*Benefits/characteristics of the allocation mechanism* (BNHCRAM) included seven measurement variables which indicated whether the allocation mechanism is less complex (Bcx4_1), meets the health care needs (Bhc4_2), improves allocative efficiency to account for different variations (Bef4_3), is an equitable method of resource allocation (Beq4_4), cater for unmet needs (Bun4_5), promotes health sector development and the economy (Bde4_6), and is generally successful (Bpe4_7).

### Regression models and SEM evaluation

SEM uses factor analysis technique to determine the number of underlying dimensions contained in a set of observed variables and to identify the subset of variables that corresponds to each of the underlying dimensions [44]. The underlying dimensions are referred to as continuous latent variables or factors. The observed variables are referred to as factor indicators. There are two types of factor analysis: exploratory factor analysis (EFA) and confirmatory factor analysis (CFA) [44–46].

SEM has basic building blocks which follow a logical sequence of five steps or processes: model specification, model identification, model estimation, model testing, and model modification. *Model specification* directly involves deciding which variables to include or not to include in the theoretical regression model as discussed in the section above. *Model identification* simply refers to deciding whether a set of unique parameter estimates can be computed for the regression equation. *Model estimation* involves estimating the parameters in the regression model—that is, computing the sample regression weights for the independent predictor variables (See S3 Table for equations summary of the measurements). *Model testing* involves determining the fit of the theoretical model. If the fit of the implied theoretical model is not as strong as one would like, then the final step is to modify the model and subsequently evaluate the new modified model [44]. However, in a confirmatory factor model approach, we seek to investigate whether the established dimensionality and factor-loading pattern fits *a priori* model—that is, whether the sample data confirm the model—then tests the hypothesis statistically. The hypothesized model is defined beforehand based on theory and/or previous analytic research. Therefore, the relationships between latent variables in the path diagram were specified as in the following equations:

$$\text{PPIAM}=\beta_{0}+\beta_{1}\text{BNHCRAM}$$ (1)

$$\text{PNIAM}=\beta_{0}+\beta_{2}\text{BNHCRAM}$$ (2)

$$\text{OPS}=\beta_{0}+\beta_{3}\text{PPIAM}+\beta_{4}\text{PNIAM}+\beta_{5}\text{BNHCRAM}$$ (3)

$$\text{ARAM}=\beta_{0}+\beta_{6}\text{PPIAM}+\beta_{7}\text{OPS}+\beta_{8}\text{PNIAM}+\beta_{9}\text{BNHCRAM}$$ (4)

Finding a statistically significant hypothetical model that has a concrete and fundamental connotation is the main goal of using SEM to test models. The following three criteria in judging the statistical significance and substantive meaning of a theoretical model are majorly used: The model-fit indices, absolute and incremental; the statistical significance of individual parameter estimates for the paths in the model; and finally, the magnitude and direction of the parameter estimates, paying particular attention to whether a positive or a negative coefficient makes sense for the parameter estimate [44].

It is suggested that several model-fit criteria be used in combination to assess the model fit which falls under three categories: absolute fit, comparative or incremental fit, and fit adjusting for model parsimony [47]. Absolute fit indices determine how well an *a priori* model fits the sample data [48] and demonstrates which proposed model has the most superior fit. These measures provide the most fundamental indication of how well the proposed theory fits the data. Unlike incremental fit indices, absolute fit indices do not rely on comparison with a baseline model, but is instead a measure of how well the model fits in comparison to no model at all [49]. On the other hand, incremental fit indices, also known as comparative or relative fit, are a group of indices that do not use the χ^2^ in its raw form but compare the χ^2^ value to a baseline model. For these models the null hypothesis is that all variables are uncorrelated [48]. The parsimony correction indices differ from absolute fit measures as they incorporate a penalty function for poor model parsimony.

Even though different authors have noted different levels of fit criteria, the traditional measure for evaluating the overall model fit is the χ^2^ value since it ‘assesses the magnitude of the discrepancy between the sample and fitted covariance matrices [50]. A good model fit would provide an insignificant result at a 0.05 threshold [51]. But, there exist limitations in the use of χ^2^ since it assumes multivariate normality and severe deviations from normality may result in model rejections even when the model is properly specified [52]. Moreover, it is sensitive to sample size where it nearly always rejects the model when large samples are used [49].

The Root-mean-square error of approximation (RMSEA) tells us how well the model with unknown but optimally chosen parameter estimates would fit the population covariance matrix [53]. However, it is sensitive to the number of estimated parameters in the model and favors parsimony in that it will choose the model with the lesser number of parameters. Traditionally, RMSEA below 0.08 shows a good fit [54]. A cut-off point of 0.90 has been recommended traditionally for the Goodness-of-fit index (GFI) but given the sensitivity of this index, it has become less popular in recent years and it has even been recommended that this index should not be used [55].

Normed fit index (NFI) compares the χ^2^ value of the model to the χ^2^ of the null model. Traditionally, NFI ≥ 0.90 has been recommended to indicate a good fit though, a major drawback to this index is its sensitivity to small sample size [56] and thus is not recommended to be solely relied on [57]. This problem was rectified by the Non-normed fit index (NNFI)/Tucker-Lewis which is an index that prefers simpler models [58]. However, Comparative fit index (CFI) is a revised form of the NFI which takes into account sample size [53], and performs well even when the sample size is small [58], and a value of CFI ≥ 0.95 is presently recognized as indicative of good fit [50].

### Analytical procedure

In the first stage, reliability analysis was performed to evaluate the stability and consistency of measured items using SPSS version 18, and variables with items-total correlation greater than 0.30 were retained in the study [41]. Data were then screened for univariate and multivariate normality and outliers using LISREL version 8.72 [59]. Since the maximum of data was at least three times bigger than the minimum, log transformation to correct the problems of skewed data, outliers, and unequal variation, was performed before a separate analysis was done [60]. We found that there was no impact on the overall result with the outliers included in the final analysis. The raw data were then prepared for analysis in the second stage.

In the second stage, a CFA using Robust Maximum Likelihood estimation procedure was performed on the constructs by seeking to statistically test the significance of the hypothesized factor model [44]. Every free parameter in the multiple regression equation was estimated from a variance-covariance matrix. This analysis was performed using LISREL version 8.72 which is the most useful tool used in SEM [49].

## Results

Table 1 presents the characteristics of survey respondents. Almost half of the respondents were female and nearly a half were under 30 years of age, and over a half had worked for more than 6 years. The leading number of specialties who responded were the nurses at 36%, followed by the physicians who were a quarter of the respondents while clinical officers accounted for 17% of the respondents. Moreover, 97.3% of the respondents in Kenya indicated that they were religious with Christianity leading at 95.9%.

**Table 1:** Characteristics of health professionals who responded to the survey.

Variables	*n* = 300	%
**Gender**		
Male	156	52.0
Female	144	48.0
**Age groups (range), years**	**(25–56)**	
≤ 30	148	49.3
31–40	108	36.0
41–50	28	9.3
≥ 51	16	5.3
**Years of experience**		
≤ 5	144	48.0
6–10	80	26.7
11–15	37	12.0
16–20	20	6.7
≥ 21	19	6.3
**Specialty**		
Physician	74	24.7
Clinical officers	51	17.0
Nurses	110	36.7
Pharmacist	20	6.7
Nutritionist	10	3.3
Others	35	11.7
Religious status		
Non-believer	8	2.7
Believer	292	97.3
**Religion (believers only)**		
Christianity	280	95.9
Islamic	10	3.4
Other	2	0.7

### Evaluation of proposed model

#### Model fit indices

Reporting the findings in this study followed the most current recommendation of reporting CFA which recommended reporting the findings of model fit indices, measurement model and structural model, only model modification indices are not reported in this study [61].

The model fit indices in Table 2 showed an interesting result with the χ^2^ being statistically significant and RMSEA is above the recommended level of 0.05. This signifies a bad fit. However, other absolute fit indices (GFI, AGFI and RMR), were high and leaning towards a perfect fit. The incremental fit indices, on the other hand, were low [48].

**Table 2:** Model fit criterion and indices for the structural equation model.

Model fit criterion	Acceptable level*	Model fit indices
**Absolute Fit Indices:**		
χ^2^ (*df*)	Compare with *df*	761 (290)
*p*-value		<. 001
Goodness-of-fit index (GFI)	0 (no fit) to 1 (perfect fit)	0.84
Adjusted GFI (AGFI)	0 (no fit) to 1 (perfect fit)	0.80
Root-mean-square error of approximation (RMSEA)	<. 05	0.16
Root-mean-square residual (RMR)	Researcher defines level	0.69
Standardized RMR (SRMR)	<. 08	0.09
**Incremental Fit Indices:**		
Normed fit index (NFI)	0 (no fit) to 1 (perfect fit)	0.28
Non-normed fit index (NNFI)/Tucker-Lewis	0 (no fit) to 1 (perfect fit)	0.22
Comparative fit index (CFI)	0 (no fit) to 1 (perfect fit)	0.30
Incremental fit index (IFI)	0 (no fit) to 1 (perfect fit)	0.31
Parsimony GFI	0 (no fit) to 1 (perfect fit)	0.69

* Acceptable level of model-fit is according to Schumacker & Lomax, 2012 recommendations.

#### Measurement variables analysis

**Fig 2:**
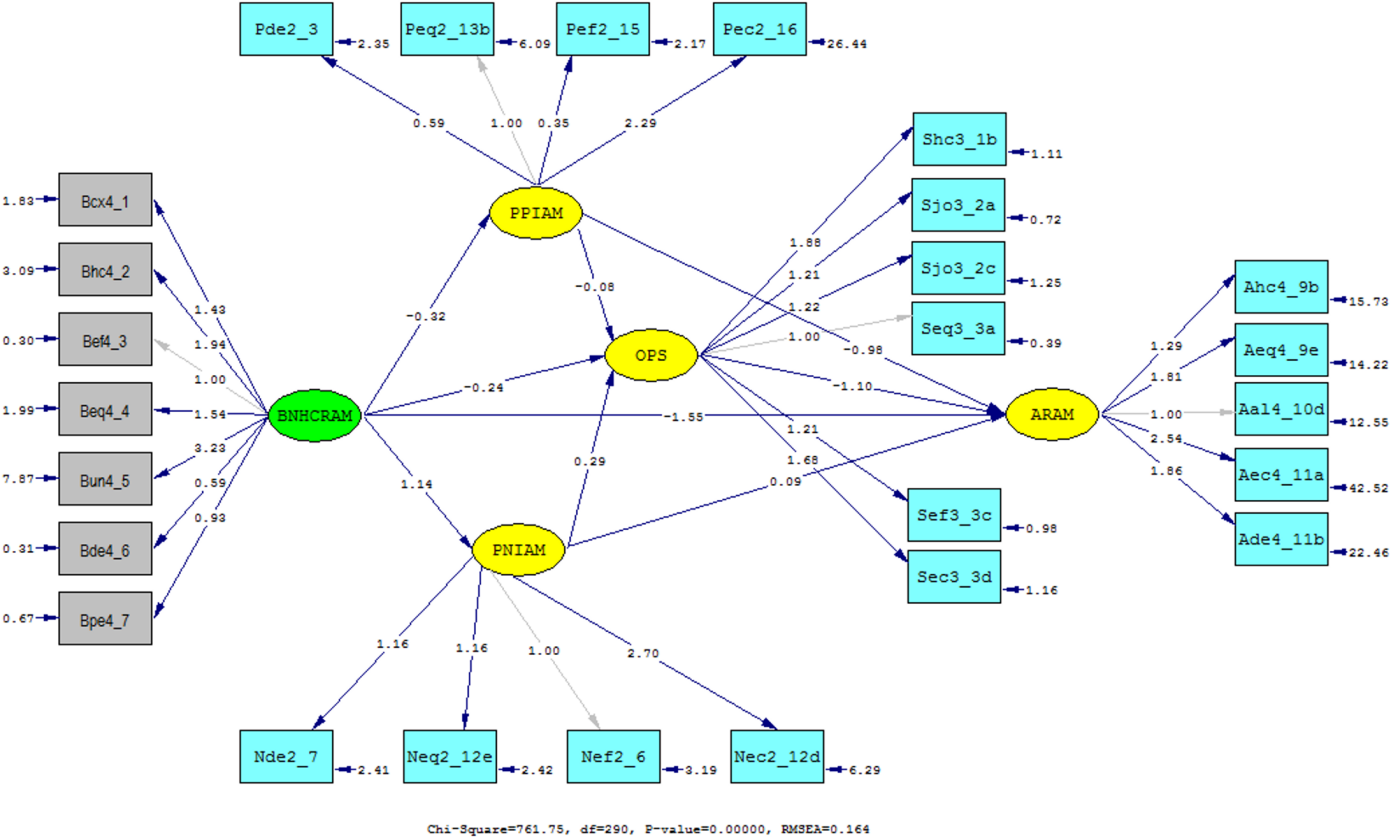
Parameter estimates of the hypothetical structural model. Path diagram generated by Lisrel 8.72

Table 3 indicates that the parameter estimates of the measured variables were all significant at *p*-value <. 001, and this showed that the covariance matrix converged, and that each measured variable contributed significantly to their respective latent construct. Therefore, path analysis was ready for interpretation. Fig 2 shows the parameter estimates of the full SEM as estimated by LISREL. Each of the observed variables is displayed in a rectangular shape, and each of the latent is displayed in an oval shape.

**Table 3:** Parameter estimates of measurement variables.

Codes	Indicator	Factor loading	Std. error	*t*-value (N = 300)
**PPIAM**	**Perceived positive impact**			
Peq2_13b	Fair distribution of resources	0.63	0.04	17.14***
Pde2_3	Acquisition of modern equipment	1.00	0.38	16.07***
Pef2_15	Increased human resource	0.35	0.04	9.59***
Pec2_16	Improved income and living standard	2.29	0.14	16.68***
**PNIAM**	**Perceived negative impact**			
Nef2_6	Increased hospital length of stay	1.16	0.10	11.62***
Nde2_7	Inadequate facilities	1.16	0.10	11.95***
Neq2_12e	Increased cost of care	1.00	0.18	17.42***
Nec2_12d	Inability to attain universal coverage	2.70	0.23	11.51***
**OPS**	**Overall Professional satisfaction**			
Shc3_1b	Patient services	1.88	0.11	17.41***
Sjo3_2a	Resource in departments	1.21	0.07	18.32***
Sjo3_2c	Necessary information	1.22	0.09	12.94***
Seq3_3a	Cost of services	1.00	0.02	16.52***
Sef3_3c	Hospital performance	1.21	0.07	16.52***
Sec3_3d	Income and standards of living	1.68	0.06	28.84***
**ARAM**	**Attitude on resource allocation**			
Ahc4_9b	Patient volume consideration	1.29	0.10	13.04***
Ade4_11b	Considers facilities and equipment	1.81	0.11	17.23***
Aec4_11a	Human resource consideration	1.00	0.57	21.89***
Aeq4_9e	Services consideration	2.54	0.15	17.14***
Aal4_10d	Hospital size consideration	1.86	0.13	14.30***
**BNHCRAM**	**Benefits of allocation mechanism**			
Bcx4_1	Less complex	1.43	0.08	17.83***
Bhc4_2	Meets health care needs	1.94	0.11	18.12***
Bef4_3	Improves allocation efficiency	1.00	0.02	13.40***
Beq4_4	Improved equity	1.54	0.10	15.57***
Bun4_5	Caters for unmet needs	3.23	0.19	17.36***
Bde4_6	Promotes development and economy	0.59	0.04	13.46***
Bpe4_7	Improves general performance	0.93	0.05	17.07***

*** *p* <. 001 (two-tailed)

#### Path analysis

Table 4 indicates the summary of the significant path hypotheses. The path hypothesis 1 (i.e. ‘benefits of national health care resource allocation mechanism’ have positive effects on ‘perceived positive impacts of allocation mechanism’) had a negative coefficient of -1.04 with an optimal level of significance at *t*(300) = -4. 71, *p* <. 001 while path hypothesis 2 (i.e. ‘benefits of national health care resource allocation mechanism’ have negative effects on ‘perceived negative impacts of allocation mechanism’) had a positive coefficient of 1.14 with a high significance level of *t*(300) = 9.18, *p* <. 001.

**Table 4:** Estimated model parameters of the full structural equation model (Eqs 1–4).

No.	Path	Parameter	Coef. (Std err)	*t*-value
H1+	BNHCRAM → PPIAM	*β*_1_	-1.04 (0.22)	-4.71***
H2-	BNHCRAM → PNIAM	*β*_2_	1.14 (0.12)	9.18***
H3+	PPIAM → OPS	*β*_3_	-0.08 (0.02)	-3.98***
H4-	PNIAM → OPS	*β*_4_	0.29 (0.05)	5.83***
H5+	BNHCRAM → OPS	*β*_5_	-0.24 (0.09)	-2.81**
H6+	PPIAM → ARAM	*β*_6_	-0.98 (0.09)	-11.23***
H7-	OPS → ARAM	*β*_7_	-1.10 (0.29)	-3.84***
H8-	PNIAM → ARAM	*β*_8_	0.08 (0.17)	0.53
H9+	BNHCRAM → ARAM	*β*_9_	-1.55 (0.29)	-5.32***

*p <. 05

**p <. 01

***p <. 001. Abbreviations: Coef., coefficient; Std. Err., standard error.

The path hypothesis 3 (i.e. ‘perceived positive impacts of allocation mechanism’ have positive effects on ‘overall health professionals’ satisfaction’) had a negative coefficient of -0.078 with a significant level of *t*(300) = -3.98, *p* <. 001.

Moreover, the path hypothesis 4 (i.e. ‘perceived negative impacts of allocation mechanism’ have negative effects on ‘overall health professionals’ satisfaction’) had a positive coefficient of 0.29 for Kenya with a highly significant level of *t*(300) = 5.83, *p* <. 001 while path hypothesis 5 (i.e. ‘benefits of national health care resource allocation mechanism’ have positive effects on ‘overall health professionals’ satisfaction’) had a negative coefficient of -0.24 with a significant level of *t*(300) = -2.81, *p* <. 01.

Path hypothesis 6 (i.e. ‘perceived positive impacts of allocation mechanism’ have positive effects on ‘attitudes on resource allocation mechanism’) had a negative coefficient of -0.98 with a very highly significant level of *t*(300) = -11.23, *p* <. 001 as well as path hypothesis 7 (‘Overall health professionals’ satisfaction’ have negative effects on ‘attitudes on resource allocation mechanism’) which had a negative coefficient of -1.10 with a significant level of *t*(300) = -3.84, *p* <. 001.

**Fig 3:**
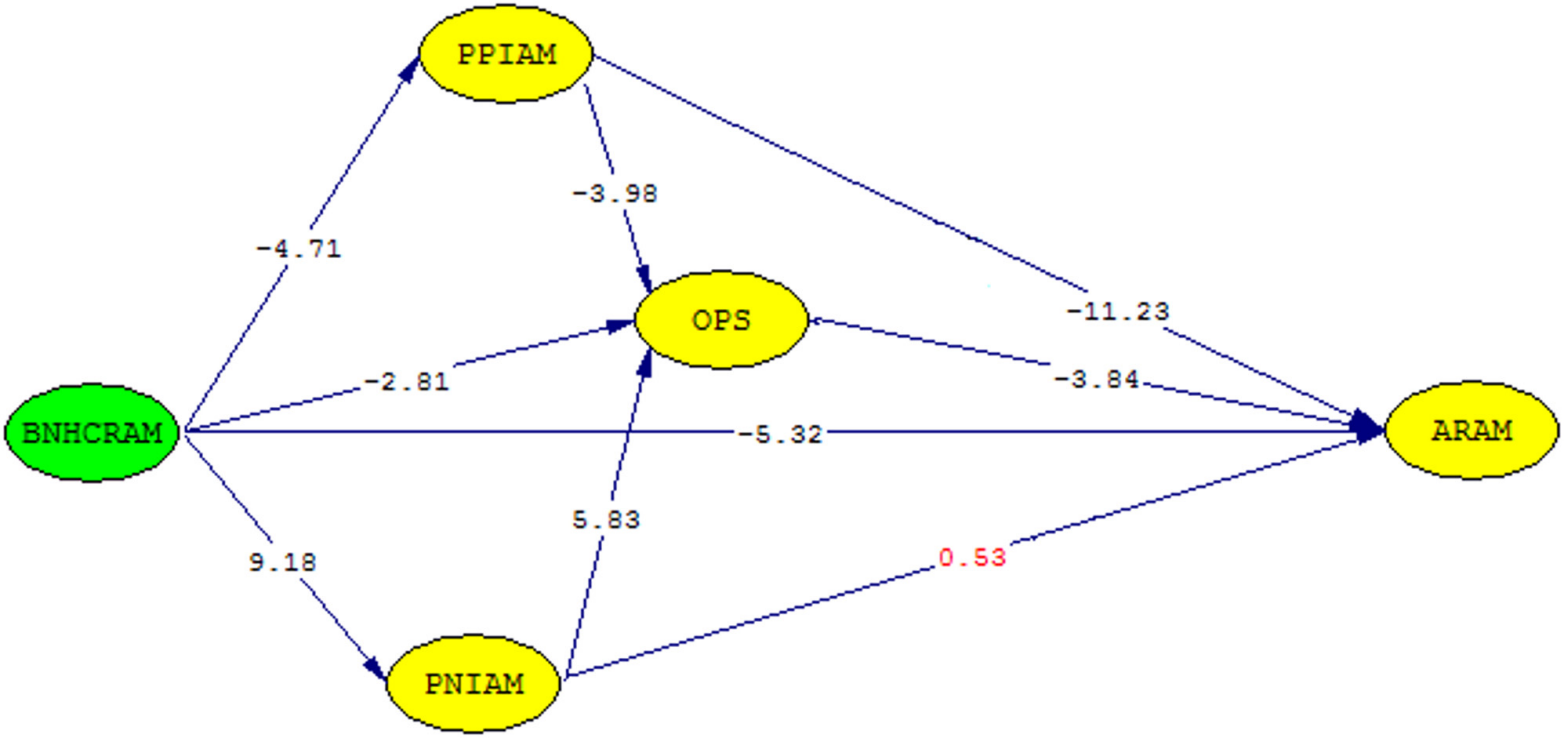
Structural model showing significant path hypotheses in *t*-values (Path value appearing in red is not significant). Path diagram generated by Lisrel 8.72

However, the path hypothesis 8 (i.e. ‘perceived negative impacts of allocation mechanism’ have negative effects on ‘attitudes on resource allocation mechanism’) had a positive coefficient of 0.088 but was not significant at *t*(300) = 0.53 while the path hypothesis 9 (i.e. ‘benefits of national health care resource allocation mechanism’ have positive effects on ‘attitudes on resource allocation mechanism’) which had a negative coefficient of -1.55 was highly significant at *t*(300) = -5.32, *p* <. 001. Fig 3 is the final model with *t*-values indicated (The path with value in red is not significant).

## Discussion

There is a general agreement amongst health professionals in MTRH that the health care resource allocation mechanism has fewer benefits which causes negative effects on the perceived positive impacts, satisfaction and attitudes. These findings in Kenya on path hypotheses indicate disgruntled professionals. The method used is clearly not the only relevant factor in the debate over the allocation of health care resources; health professionals, institutions, political, social, and economic factors play a large role as well. Health professionals have limited resources and must work within constraints to achieve the best outcome possible.

Health professionals in MTRH are in agreement that there are many negative impacts associated with the resource allocation mechanism. This means that the fewer benefits associated with the allocation mechanism used in Kenya seem to be outweighed by the negative effect perceived on development, equity, efficiency and the economy. It would therefore suggest that the relationships of the allocation criteria and health professional attitude in Kenya be further investigated to understand if other indicators may be more reflective of these associations.

Health professionals are in agreement that the allocation mechanism has positive effects on negative impact. This result observed comes at a time the government increased its total budget by 9.9% from the year 2009/10 to 2010/11 and reduced the proportion allocated to the health sector from 7.0% in 2009/10 to 6.5% in 2010/11 [62], falling further below the Abuja declaration target of increasing health spending to 15% of the government spending. Moreover, given the context of this study in Kenya, the health sector was marred with a lot of strikes from its professionals which therefore would have led the majority of respondents to be chagrined with the resource allocation mechanism to which would have increased the negative effects in the economy and health system [25].

Maintaining the status quo has two significant implications in Kenya. First, health professionals will continue feeling dissatisfied with their health system and have a contrariness attitude and thus a negative effect on the sick population and subsequently the economy. Secondly, the integrity of the health system may not be maintained leading to a market driven health system which would further hurt the poor population which is a greater percentage in the country. The effects of resource allocation mechanism also have the effect of escalating cost of care and unequal distribution of the limited resources.

The negative effects on health professionals’ satisfaction in Kenya indicate serious consequences on the satisfaction of the health care, job, equity, efficiency and the economy. This is in agreement with the low satisfaction level of staff with the health ministry’s work to provide opportunities for growth [63]. This indicates that health professionals who are dedicated in providing services to the population may not be performing their duties to the utmost. This can, therefore, lead to unproductivity, worker turnover, worker conflict, absenteeism, higher health care costs and subsequently inequity and overall disease burden and problems which make even work stress a $200 billion a year concern for organizations [64, 65]. According to the U.K. Health and Safety Executive recent publication, the occupation that reported the highest rates of total cases of work-related stress, depression or anxiety (three-year average) were health professionals [66].

Phelps [67] noted that health professionals are an important input in the production of medical care, and that the only primary way by which hospitals attract them to their staffs is by providing the capacity for them to do things they cannot do elsewhere. When it is necessary for physicians to decide the kind of medical services to offer, they can make better choices at a reasonable cost since they are the patients’ agents in deciding which treatment is appropriate. Rice [68] also indicated that there are always little progress in developing systems that both encourage health professionals to act as good agents towards society by controlling costs as well as being good agents to patients by providing high quality care.

As the Kenyan way of governance is being restructured in the name of efficiency and effectiveness, trust in the health care allocation mechanism is becoming an increasingly important component in determining health system and employee performance. Trust in the system also has a significant impact on other factors such as perceived fairness of decisions [69], job satisfaction [70–72], the quality of work life, and organizational and clinical effectiveness [73–75]. It is quite probable that mistrust of the system could potentially threaten the quality of care to patients.

The negative effect on attitude exhibited in the model is, however, in disagreement with the model of Ko and Stewart, who found a positive relationship between benefits and perceived positive impacts with attitude in a different sector [35]. It is interesting to note that health professionals’ negative attitude towards allocation mechanism was reflected in several strikes from the year 2011 to 2013 in Kenya, which led to several professionals to quitting government service [25]. Studies have found that health professionals’ perceptions and attitudes are influenced by both internal (e.g. personal characteristics) and external factors (e.g. attitude of the organization for which they work) [76–78]. The negative effect of the allocation mechanism as revealed by this study indicate that health professionals have much higher expectation, even as Kenya implements its new constitution that will see a new system of governance in the health sector. It would be necessary if further investigation is done, to enable the policymakers to identify areas of improvement in the new devolved structure of governance.

In 2000, WHO encouraged nations to aim at maintaining financial protection (ensuring that people do not become poor as a result of using health care) and equitable distribution of health [79]. However, this can only be achieved by first involving all the stakeholders, especially health professionals who understand the health care system at the frontline. Studies have mostly been conducted on to understand perceptions and attitudes of health professionals on health system reforms in different countries [78, 80–84] but unlike all of them, this study attempted to understand perceptions and attitudes health care resource allocation.

### Conclusion

#### Policy implication

In conclusion, this study is one of the first to investigate the perceptions and attitudes of health professionals on health care resource allocation in Kenya toward addressing the inequitable distribution of health care resources in Sub-Saharan Africa. We analyzed perceptions and attitudes of health professionals and observed that health care service providers have a negative opinion regarding resource allocation. Two points are worth highlighting. First, our results indicated that even though Kenya had been using MBC and RAC in the allocation of health care resources at national and district levels, respectively, there are challenges that are closely concomitant with the allocation mechanism. In the context of Kenya, our findings clearly demonstrate how health care resource allocation strategy is associated with perceptions and attitudes of health professionals in a national hospital in Kenya. Kenya has two national hospitals, Kenyatta National Hospital and MTRH, but MTRH was adversely affected by the strikes and subsequent resignation of its health professionals. The negative relation emphasizes the role of availability of resources towards equity, development and efficiency, and job satisfaction. Equitable distribution of resources not only improves availability of health care services to the populace, but may also increase the satisfaction of health professionals in public service, who are currently seeking to offer their services elsewhere. This may help to narrow the disparities in health care service to the people, by allowing providers to give appropriate and timely services they need to offer. These findings may clarify policy debates on the realization of equity in health care in low income countries. Second, we suggest that policymakers consider reviewing allocation strategy, and pursue greater efforts toward consideration of various regions with different needs, and more especially, needs of different health departments in collaboration with providers who are at the frontline of health care service. Achieving this may be, in part, involving different health sector departments in the allocation of health care resources and getting their views on the needs of each department. The MOH should develop the capacity to oversee and strengthen allocation strategy and management support for an enhanced information system for accurate information. An independent evaluation of resource availability and utilization in different regions and departments is required.

### Limitations and future research

Even though SEM is a complex-analytic framework, there is need of noting several problematic issues concerning the estimation and testing of individual parameters that are usually overlooked in SEM applications. First, since SEM models are approximations, it is appropriate to note that the parameter estimates and associated standard errors generated by analyses are unbiased only under the assumption that the specified model is correct. Amplifying the problem here is the phenomenon of the propagation of specification errors. The estimators most commonly used in empirical applications of SEM (e.g., maximum likelihood) use all available information in the covariance matrix of the observed variables to generate parameter estimates. While this feature is related to several advantages (e.g., smaller standard errors when models are correct), it also permits the effects of a misspecified parameter to be propagated beyond the specific equation in which it occurs [85]. Secondly, given the large number of parameters used in the study, the sample size used may also be small, and hence future studies may try a large sample size to test the model. In addition, although the test statistic assumes that the sampling distribution of a parameter is normal, it is likely that the distributions are not symmetrical given the small sample size used. Another limitation would include the inability to generalize the findings from the study since it focused only on one national hospital. Miller noted that the technical path analysis, is not a method of discovering causal laws, but a procedure for giving a quantitative interpretation of an assumed theoretical causal system as it operates within a given population, and therefore, over interpretation and generalization of the findings should be avoided [86]. Future studies should therefore consider examining more than one hospital and at different levels of the health system. Finally, Box also noted that all models are wrong, but some are useful [87]. However, the findings of this study can serve as an essential reference for policymakers regarding the health care resource allocation and priority setting in Sub-Saharan Africa, such as Kenya. Moreover, for practical and descriptive purposes, this study provides a valuable starting point of discussion. The health sector and resource allocation are important in the globe today, and it is therefore imperative that national health care resource allocation receives more attention as partly supported by the findings presented here. Future research could therefore look at the same area of study using a different approach that will extend the findings of this study. Also, the negative association found between the allocation mechanism and attitudes of health professionals should be explored further to understand if there are other confounding factors that may result to this effect, and to give more insight on how to improve the allocation strategy in the new devolved system of governance.

## Supporting Information

S1 Table Weighted variables in Kenya’s Resource Allocation Criteria formula. (PDF)

S2 Table Reliability analysis of measurement variables in the five latent constructs. (PDF)

S3 Table Summary of the equations showing relationship between measurement variables and the latent variables. (PDF)

S1 Appendix The final survey questionnaire. (PDF)
