# Supplementary material for: Perceptions and Attitudes of Health Professionals in Kenya on National Health Care Resource Allocation Mechanisms: A Structural Equation Modeling
Source: PLoS One. 2015 Jun 3;10(6):e0127160. doi: 10.1371/journal.pone.0127160 (PMC4454489; doi:10.1371/journal.pone.0127160)
Supplement: S1 Table — (PDF) [file pone.0127160.s001.pdf]

S1 Table: Weighted variables in Kenya's Resource Allocation Criteria formula

**Variable**	**District Hospitals**	**Rural Health Facilities**
	Weight	*Weight*
Bed use	0.40	-
Poverty rate	0.20	0.30
Outpatient case load	0.20	-
Fuel costs	0.15	-
Accident area	0.05	-
Under-5 population	-	0.20
Females of reproductive age (15 – 49 years)	-	0.20
AIDS cases	-	0.05
Infrastructure	-	0.15
Area of district (Km^2)	-	0.10
Total	1.00	1.00
