# Supplementary material for: Perceptions and Attitudes of Health Professionals in Kenya on National Health Care Resource Allocation Mechanisms: A Structural Equation Modeling
Source: PLoS One. 2015 Jun 3;10(6):e0127160. doi: 10.1371/journal.pone.0127160 (PMC4454489; doi:10.1371/journal.pone.0127160)
Supplement: S2 Table — (PDF) [file pone.0127160.s002.pdf]

S3 Table: Reliability analysis of measurement variables in the five latent constructs

Codes	Indicator	Mean (SD)	Item-total correlation	α-If item is deleted
**PPIAM**	**Perceived positive impact (Cronbach-α)**			**(0.80)**
Peq2_13b	Fair distribution of resources	1.8 (1.3)	0.65	0.73
Pde2_3	Acquisition of modern equipment	2.2 (1.4)	0.62	0.75
Pef2_15	Increased human resource	2.0 (1.2)	0.62	0.75
Pec2_16	Improved income and living standard	1.8 (1.3)	0.57	0.77
**PNIAM**	**Perceived negative impact (Cronbach-α)**			**(0.82)**
Nef2_6	Increased hospital length of stay	4.3 (0.8)	0.58	0.81
Nde2_7	Inadequate facilities	3.9 (0.8)	0.63	0.80
Neq2_12e	Increased cost of care	4.1 (1.1)	0.73	0.74
Nec2_12d	Inability to pool finances	3.4 (1.5)	0.81	0.73
**OPS**	**Overall Professional satisfaction (Cronbach-α)**			**(0.85)**
Shc3_1b	Patient services	1.3 (0.5)	0.68	0.83
Sjo3_2a	Resource in departments	1.4 (0.6)	0.59	0.84
Sjo3_2c	Necessary information	1.5 (0.8)	0.57	0.84
Seq3_3a	Cost of services	1.6 (0.7)	0.73	0.81
Sef3_3c	Hospital performance	1.6 (0.8)	0.63	0.83
Sec3_3d	Income and standards of living	1.6 (0.8)	0.75	0.81
**ARAM**	**Attitude on resource allocation (Cronbach-α)**			**(0.86)**
Ahc4_9b	Patient volume consideration	4.1 (1.2)	0.61	0.84
Ade4_11b	Considers facilities and equipment	3.5 (1.5)	0.76	0.81
Aec4_11a	Human resource consideration	3.2 (1.7)	0.73	0.81

Codes	Indicator	Mean (SD)	Item-total correlation	α-If item is deleted
Aeq4_9e	Services consideration	3.0 (1.6)	0.63	0.83
Aal4_10d	Hospital size consideration	3.2 (1.6)	0.61	0.84
**BNHCRAM**	**Benefits of allocation mechanism (Cronbach-α)**			**(0.82)**
Bcx4_1	Less complex	1.3 (0.6)	0.49	0.81
Bhc4_2	Meets health care needs	1.5 (0.8)	0.54	0.80
Bef4_3	Improved allocation efficiency	1.6 (0.7)	0.76	0.76
Beq4_4	Improved equity	1.7 (0.9)	0.55	0.80
Bun4_5	Cater for unmet needs	1.7 (1.0)	0.67	0.78
Bde4_6	Promotes development and economy	1.4 (0.5)	0.48	0.81
Bpe4_7	Improves general performance	1.3 (0.6)	0.54	0.80
