# Supplementary material for: Perceptions and Attitudes of Health Professionals in Kenya on National Health Care Resource Allocation Mechanisms: A Structural Equation Modeling
Source: PLoS One. 2015 Jun 3;10(6):e0127160. doi: 10.1371/journal.pone.0127160 (PMC4454489; doi:10.1371/journal.pone.0127160)
Supplement: S3 Table — (PDF) [file pone.0127160.s003.pdf]

S4 Table: Summary of the equations showing relationship between measurement variables and the latent variables

Latent Construct =	*Measurement Variables*
Perceived positive impacts of allocation mechanism =	β_0 + β_1 Fair distribution of resources + β_2 Acquisition of modern equipment + β_3 Increase in human resource + β_4 Improved income and standards of living
Perceived negative impacts of allocation mechanism =	β_0 + β_1 Increased cost of care + β_2 Inadequate facilities + β_3 Increased hospital length-of-stay + β_4 Inability to attain universal coverage
Overall health professionals' satisfaction =	β_0 + β_1 Patients' services offered + β_2 Resources available + β_3 Information available + β_4 cost of services + β_5 hospital performance + β_6 income and standards of living
Attitude on allocation of health care resources =	β_0 + β_1 Consider patient volume + β_2 Improvement on facilities and equipment + β_3 Increase in human resource + β_4 Patients' service restrictions + β_5 hospital size
Benefits or characteristics of the allocation mechanism =	β_0 + β_1 Less complex + β_2 Meets the health care needs + β_3 Improves allocative efficiency to account for different variations + β_4 An equitable method of resource allocation + β_5 Cater for unmet needs + β_6 Promotes health sector development and economy + β_7 Generally successful
