# Supplementary material for: Perceptions and Attitudes of Health Professionals in Kenya on National Health Care Resource Allocation Mechanisms: A Structural Equation Modeling
Source: PLoS One. 2015 Jun 3;10(6):e0127160. doi: 10.1371/journal.pone.0127160 (PMC4454489; doi:10.1371/journal.pone.0127160)
Supplement: S1 Appendix — (PDF) [file pone.0127160.s004.pdf]

PERCEPTIONS ON NATIONAL HEALTH CARE RESOURCE ALLOCATION MECHANISM

SURVEY QUESTIONNAIRE TO HEALTH PROFESSIONALS

Dear Health Professionals,

The national health care resources are allocated, but health professionals are still faced with challenges caused by the allocation mechanism. Due to limited medical resources, health professionals occupy a position which makes them notice the impact of changes set by the health care system and as a result they are confronted with the effects of health care resource allocation mechanism on their clinical practice.

The purpose of this survey is to give an understanding of the national health care resource allocation mechanism from the health professionals' perspective, and this will give a useful insight regarding priority setting. We hope that through this questionnaire, health professionals' views will be brought into light, and this will enhance proper distribution of health care resources. Thank you for your contribution in the health care system.

We are going to ask your opinion but your answers will be **completely confidential** and your name will never be used in connection to the information you give. The survey will take approximately **10 minutes** and we will appreciate if you sign here as a sign of willingness to participate:

Name:__________________________________ Sign:________________________ Date:_____________

PART I: - PERSONAL INFORMATION ***(Please mark appropriately with ☑)***

1. Year of birth: ____________ **2**. Years of experience: ___________ **3**. Sex: ☐ Male ☐ Female

4. Health professionals: ☐ Physician ☐ C/O ☐ Nurse ☐ Pharmacist ☐Nutritionist ☐Other:______

5. Religious status: ☐ Non-believer ☐ Believer： ☐ Christian ☐ Islamic ☐ Other______

PART II: PERCEIVED IMPACT OF HEALTH CARE RESOURCE ALLOCATION ***(Please mark with ☑)***

**No.**	**Question**	**Strongly Disagree**	**Disagree**	**Sometimes**	**Agree**	**Strongly Agree**
1.	In the last 6 months there was an increase in the number of outpatients you attended to.	**1**	**2**	**3**	**4**	**5**
2.	In the last 6 months there was an increase in the number of inpatients you attended to.	**1**	**2**	**3**	**4**	**5**
3.	In the last 6 months the hospital acquired modern equipment you needed for diagnosis and treatment of your patients.	**1**	**2**	**3**	**4**	**5**
4.	In the last 6 months you worked extra hours a day due to the increased number of patients.	**1**	**2**	**3**	**4**	**5**
5.	In the last 6 months you attended to patients with a case of medical error.	**1**	**2**	**3**	**4**	**5**
6.	In the last 6 months you encountered some patients who have stayed in the inpatient unit for more than two weeks to receive medical care.	**1**	**2**	**3**	**4**	**5**
7.	In the last 6 months you were sometimes unable to obtain some services you thought were necessary for your patients (including unacceptable waiting time)	**1**	**2**	**3**	**4**	**5**
8.	In the last 6 months sometimes you felt under pressure to deny an expensive intervention to your patients.	**1**	**2**	**3**	**4**	**5**
9.	In the last 6 months you found in your work that some patients have problems that cannot be treated because they cannot afford their share of costs.	**1**	**2**	**3**	**4**	**5**
10.	In the last 6 months you saw situations where a patient suffered adverse consequences as a result of limited resources in the health care.	**1**	**2**	**3**	**4**	**5**

11.	**What is the most severe adverse consequence you have seen as a result of limited resources in health care?** (Can tick more than 1) ① *Inconvenience* ② *Temporary disability* ③ *Permanent disability* ④ *Acute life-threatening event* ⑤ *Death* ⑥ *None*					
12.	**Based on your experience, which patients are more likely than others to be denied beneficial care on the basis of cost in your health care environment?**					
	**a)** At least one age group is likely to be denied beneficial care on the basis of cost.	**1**	**2**	**3**	**4**	**5**
	**b)** Mentally incapacitated patients are likely to be denied beneficial care on the basis of cost.	**1**	**2**	**3**	**4**	**5**
	**c)** Those who require chronic care are likely to be denied beneficial care on the basis of cost.	**1**	**2**	**3**	**4**	**5**
	**d)** Those without health insurance are likely to be denied beneficial care on the basis of cost.	**1**	**2**	**3**	**4**	**5**
	**e)** Those who cannot pay for treatment are likely to be denied beneficial care.	**1**	**2**	**3**	**4**	**5**
	**f)** Those who need expensive treatment are more likely to be denied beneficial care.	**1**	**2**	**3**	**4**	**5**
13.	**Based on your experience, please indicate your view on the following:**					
	a) I am given enough means to care my patients fairly.	**1**	**2**	**3**	**4**	**5**
	b) Health care resources in my country are distributed fairly.	**1**	**2**	**3**	**4**	**5**
	c) Everyone in my country has equal access to needed medical services.	**1**	**2**	**3**	**4**	**5**
14.	Health care equipment and supplies are available for appropriate care of your patients in your hospital.	**1**	**2**	**3**	**4**	**5**
15.	Employment of fulltime physicians and other health care personnel has increased.	**1**	**2**	**3**	**4**	**5**
16.	Since you started working your income and living standards have improved.	**1**	**2**	**3**	**4**	**5**
17.	Some other departments have more than what they need as compared to your department.	**1**	**2**	**3**	**4**	**5**
18.	There are some services which you feel cannot be offered and are to be sought for elsewhere.	**1**	**2**	**3**	**4**	**5**

PART III – OVERALL HEALTH PROFESSIONAL'S SATISFACTION

**No.**	**Question**	**Strongly Disagree**	**Disagree**	**Sometimes**	**Agree**	**Strongly Agree**
**1.**	**In your opinion do you think you are satisfied with health care service to the following:**					
	**a)** Health care service to all emergency cases is satisfactory.	**1**	**2**	**3**	**4**	**5**
	**b)** Health care service to all outpatients is satisfactory.	**1**	**2**	**3**	**4**	**5**
	**c)** Health care service to all inpatients is satisfactory.	**1**	**2**	**3**	**4**	**5**
	**d)** A health care service to all long-term care patients is satisfactory.	**1**	**2**	**3**	**4**	**5**
**2.**	**In your opinion do you think you are satisfied with the following areas related to your job:**					
	**a)** How health care resources are distributed to serve your professional need is satisfactory.	**1**	**2**	**3**	**4**	**5**
	**b)** The support given towards your professional growth is satisfactory.	**1**	**2**	**3**	**4**	**5**
	**c)** Information you have on national health care resource allocation mechanism is satisfactory.	**1**	**2**	**3**	**4**	**5**
**3.**	**In your opinion do you think you are satisfied with the following economic aspects:**					
	**a)** Availability and affordability of health care services to all is satisfactory.	**1**	**2**	**3**	**4**	**5**
	**b)** Number of health professionals employed to serve the population is satisfactory.	**1**	**2**	**3**	**4**	**5**
	**c)** The general performance of the hospital is satisfactory	**1**	**2**	**3**	**4**	**5**
	**d)** Your income and standard of living is satisfactory.	**1**	**2**	**3**	**4**	**5**

PART IV: NATIONAL HEALTH CARE ALLOCATION MECHANISM AND ATTITUDE

No.	Question	Strongly Disagree	Disagree	Sometimes	Agree	Strongly Agree
**1.**	The national health care allocation mechanism used in your country is easily understood by the health professionals.	**1**	**2**	**3**	**4**	**5**
**2.**	The national health care allocation mechanism used in your country meets health care needs and circumstances of the population and physicians.	**1**	**2**	**3**	**4**	**5**
**3.**	The national health care allocation mechanism used in your country provides some allocative efficiency to account for variations in population needs.	**1**	**2**	**3**	**4**	**5**
**4.**	The national health care allocation mechanism in your country provides an equitable method of resource allocation for variations in service volumes and case complexity.	**1**	**2**	**3**	**4**	**5**
**5.**	The national health care allocation mechanism used in your country addresses potential problems of estimating unmet population need.	**1**	**2**	**3**	**4**	**5**
**6.**	The national health care allocation mechanism used in your country caters for the improvement in health infrastructure and health professionals' living standards.	**1**	**2**	**3**	**4**	**5**
**7.**	The national health care allocation mechanism used in your country is generally successful.	**1**	**2**	**3**	**4**	**5**
**8.**	You are well informed on how health care resources are allocated and how your stipend is determined.	**1**	**2**	**3**	**4**	**5**
**9.**	**Based on your experience, how acceptable do you consider the following methods of resource allocation to be?**					
	**a)** Allocation of resources should consider the seriousness of cases.	**1**	**2**	**3**	**4**	**5**
	**b)** Allocation of resources should consider the number of patient visits (inpatient and outpatient).	**1**	**2**	**3**	**4**	**5**
	**c)** Allocation of resources should consider the number of hospital beds.	**1**	**2**	**3**	**4**	**5**
	**d)** Allocation of resources should promote the use of direct treatment fee.	**1**	**2**	**3**	**4**	**5**
	**e)** There should be restrictions on administering expensive treatment and examinations.	**1**	**2**	**3**	**4**	**5**
	**f)** Allocation of resources should be limited to specific services offered in health care facility.	**1**	**2**	**3**	**4**	**5**
**10.**	**Based on your experience, should the following be given priority than the other?**					
	**a)** Priority should be given to more serious cases than to less serious cases.	**1**	**2**	**3**	**4**	**5**
	**b)** Priority should be given to services to children than to services to senior citizens.	**1**	**2**	**3**	**4**	**5**
	**c)** Priority should be given to physical problems than to mental problems.	**1**	**2**	**3**	**4**	**5**
	**d)** Priority should be given to larger hospitals than to smaller hospitals.	**1**	**2**	**3**	**4**	**5**
	**e)** Priority should be given to preventive programs than on treatment of rare cases.	**1**	**2**	**3**	**4**	**5**
	**f)** Priority should be given to inpatients than outpatients.	**1**	**2**	**3**	**4**	**5**
**11.**	**What is your opinion on applying the following approaches to improve health care system?**					
	**a)** Employment of physicians and other health professionals should be increased.	**1**	**2**	**3**	**4**	**5**
	**b)** Improving facilities and equipment to enable administration of advanced medical services.	**1**	**2**	**3**	**4**	**5**
	**c)** Improving on the allocation mechanism to enable equitable coverage.	**1**	**2**	**3**	**4**	**5**
	**d)** Raising the income of health professionals and subsequently their living standards.	**1**	**2**	**3**	**4**	**5**

12. Please write other suggestion(s) you consider can help in improving health care system.

____

____

____
